# Synapse Innervation and Associative Memory Cell Are Recruited for Integrative Storage of Whisker and Odor Signals in the Barrel Cortex through miRNA-Mediated Processes

**DOI:** 10.3389/fncel.2017.00316

**Published:** 2017-10-25

**Authors:** Zhuofan Lei, Dangui Wang, Na Chen, Ke Ma, Wei Lu, Zhenhua Song, Shan Cui, Jin-Hui Wang

**Affiliations:** ^1^School of Pharmacy, Qingdao University, Dengzhou, China; ^2^Institute of Biophysics, Chinese Academy of Sciences, Beijing, China; ^3^Department of Biology, University of Chinese Academy of Sciences, Beijing, China

**Keywords:** learning, memory, synapse, memory cell, cortex, microRNA, Ttbk1

## Abstract

Associative learning is a common way for information acquisition, and the integrative storage of multiple associated signals is essential for associative thinking and logical reasoning. In terms of the cellular mechanism for associative memory, our studies by behavioral task and cellular imaging demonstrate that paired whisker and odor stimulations lead to odorant-induced whisker motion and associative memory cell recruitment in the barrel cortex (BC), which is driven presumably by synapse innervation from co-activated sensory cortices. To confirm these associative memory cells and synapse innervations essential for associative memory and to examine their potential mechanisms, we studied a causal relationship between epigenetic process and memory cell/synapse recruitment by manipulating miRNAs and observing the changes from the recruitments of associative memory cells and synapse innervations to associative memory. Anti-miRNA-324 and anti-miRNA-133a in the BC significantly downregulate new synapse innervation, associative memory cell recruitment and odorant-induced whisker motion, where Tau-tubulin kinase-1 expression is increased. Therefore, the upregulated miRNA-324 in associative learning knocks down Ttbk1-mediated Tau phosphorylation and microtubule depolymerization, which drives the balance between polymerization and depolymerization toward the axon prolongation and spine stabilization to initiate new synapse innervations and to recruit associative memory cells.

## Introduction

Associative memory plays important roles in cognitive processes, such as logical reasoning and associative thinking (Kandel and Pittenger, 1999; Bailey et al., 2015; Wang and Cui, 2017). Animal models of conditioned reflexes (CR) have been used to elucidate mechanisms underlying associative learning and memory (Wasserman and Miller, 1997). Activity-dependent plasticity at synapses and neurons is associated with memory formation (Bliss and Collingridge, 1993; Blair et al., 2001; Maren, 2005; Karmarkar and Dan, 2006; Holtmaat and Svoboda, 2009; Silva et al., 2009). Associative memory cells are detected in the co-activated sensory cortices (Wang et al., 2015; Gao et al., 2016; Vincis and Fontanini, 2016) and their downstream brain areas (Naya et al., 2003; Takehara-Nishiuchi and McNaughton, 2008; Viskontas, 2008; Cai et al., 2016). Although new synapse innervation may constitute one of the driving forces to recruit associative memory cells, which are associated with associative memory (Wang et al., 2015, 2016, 2017; Gao et al., 2016; Wang and Cui, 2017), the causal relationship from new synapse innervation and associative memory cell recruitment to associative memory formation needs to be examined, as well as potential mechanisms underlying new synapse formation, associative memory cell recruitment and cross-modal associative memory remain to be addressed.

As the integration and storage of associated signals are induced by environmental information, epigenetics-mediated processes may be involved (Molfese, 2011; Kaas et al., 2013; Landry et al., 2013; Lattal and Wood, 2013; Woldemichael et al., 2014). The level of some miRNAs is altered during associative memory, especially the upregulation of miRNA-324 and miRNA-133a that influence actin filaments as well as microtubules for their polymerization (Yan et al., 2016). Since the balance between microtubule polymerization and depolymerization influences tubulin growth that plays a critical role in axon prolongation and spine stabilization (Dent and Kalil, 2001; Mitsuyama et al., 2009; Wilson et al., 2012; Ueda et al., 2015), we hypothesize that associative learning induces a chain reaction of miRNA expression change, microtubule polymerization, axon prolongation and synapse innervation for associative memory cell recruitment. Here, we aim to examine how miRNA-324/miRNA-133a and their regulated molecules serve to the recruitments of new synapse innervations and associative memory cells for the integration and storage of associated signals.

In terms of strategies to address new synapse innervation and associative memory cell formation for cross-modal associative memory by epigenetic process, the blockade of miRNA upregulation was performed by injecting miRNA antagomirs into the barrel cortex (BC). The molecular targets of miRNAs were studied by using dual luciferase report assay and western-blot. The detection of associative memory cells in the BC was done by using two-photon cell imaging *in vivo*. Synapse innervations were detected by using the neural tracing with fluorescent-tagged adeno-associated virus. In addition, the rationale for studying a role of epigenetic-mediated regulation in associative memory cell recruitment in the BC was based on our previous studies that this area was a primary location for cross-modal associative memory (Wang et al., 2015, 2016, 2017; Gao et al., 2016).

## Materials and Methods

All experiments were conducted based on the guideline by Administration Office of Laboratory Animals at Beijing China. Experiment protocols were approved by Institutional Animal Care Unit Committee in Administration Office of Laboratory Animals at Beijing China (B10831).

### Mouse Model of Associative Memory

C57 Thy1-yellow fluorescent protein (YFP) mice (The Jackson Labs, USA, Feng et al., 2000) have been used in our study, in which certain amount of glutamatergic neurons was genetically labeled by YFP due to a weak promotion of Thy1 in order to analyze cell-specific mechanism for associative memory. In our study, 54 male mice were used. In general, they were accommodated in the cages (*n* = 4 mice per cage) with a usual circadian cycle about 12 h under light condition and 12 h under dark condition.

The two groups of mice in postnatal day 20 were treated by microinjecting the antagomirs of miRNA-324-5p/miRNA-133a-3p and the control of these antagomirs into barrel cortices, respectively. Forty-eight hours after the injection, these mice were trained by pairing mechanic whisker stimulus (WS) with odor stimulus (OS, butyl acetate toward their noses; Wang et al., 2015; Gao et al., 2016; Yan et al., 2016; Guo et al., 2017; Liu et al., 2017; Zhao et al., 2017). The concentration of butyl acetate was 99.99% in analytic purity. The paired WS and OS were given by a multiple-sensory modal stimulator (ZL201410499466), in which the intensities, time and intervals of OS and WS were precisely set. The OS intensity was sufficient to induce the activity of olfactory bulb neurons, and the WS intensity was sufficient to induce whisker fluctuation after WS ended. Each of the mice was trained 20 s each time, five times per day with 2 h of intervals for 10 days. During the training, each mouse was placed in a home-made cage. In entire experimental procedures, these mice were not experiencing stressful condition and circadian disturbance. In addition, the mice have normal whisking and symmetric whiskers (Wang et al., 2015; Gao et al., 2016; Yan et al., 2016; Guo et al., 2017).

Whisker motion tracks were monitored by a digital video camera (240 fps) and were quantified in onset latency from odor test to whisker motion, whisking frequency and whisking duration (the software self-programed in Matlab; and ImageJ, version. 1.47, the National Institute of Health, Rockville, MD, USA). Whisking frequency and duration were converted into digital signals in 0.5 s as one unit, such that their dynamic values per second were presented before, during and after odor-test. The responses of mouse whiskers to the odor-test (butyl acetate, 20 s) were measured at the end of each training day to quantify the onset time and levels of the CR. CR-formation was defined to meet the following criteria. The patterns of odorant-induced whisker motion were similar to those of WS-induced whisker motion. Whisking frequency and whisker retraction time were significantly increased as well as the latency of whisker motion in response to odor-test was significantly reduced, compared with control and before the training. As this type of whisker motion induced by odorant was originally induced by WS, the odor signal evoked a recall of whisker signal and led to whisker motion (Wang et al., 2015; Gao et al., 2016; Yan et al., 2016; Guo et al., 2017).

Long whiskers (such as arcs 1–2) on the same side and rows were assigned for mechanical stimulations and for the observations during the odor-test. This selection was based on the studies of cross-modal plasticity (Ni et al., 2010; Ye et al., 2012; Zhang et al., 2013). We did not trim the shorter whiskers since whisker trimming elevated the excitability of the BC (Zhang et al., 2013).

### The Injection of miRNA Antagomirs into the Barrel Cortex

**Figure 1:**
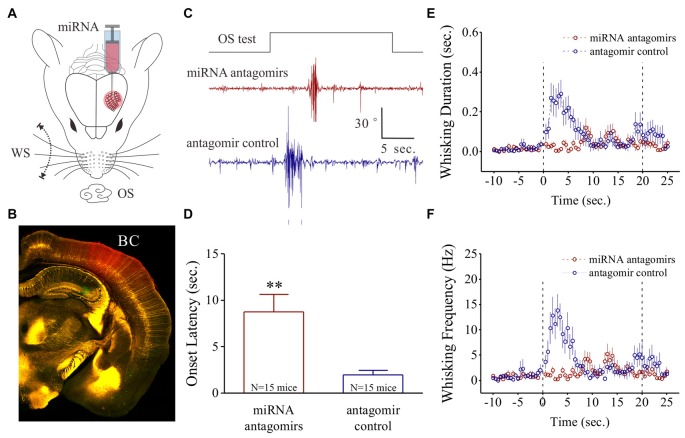
Odorant-induced whisker motion is impaired by anti-miRNA-324-5p and anti-miRNA-133a-3p. **(A)** These miRNA antagomirs and their controls were injected into the barrel cortices of two groups of the mice, respectively. The mice then were trained by pairing whisker stimulus (WS) and odor stimulus (OS) for 10 days. **(B)** After their behavioral tests, the correct injections of miRNA antagomirs (conjugated with red fluorescent) were examined in the barrel cortices. **(C)** shows the impaired whisker motions in response to OS-test from a mouse in antagomir group (red trace) and a mouse in control group (blue). Calibration bars are 30 degrees of whisker retraction and 5 s. **(D)** Latencies from OS onset to whisker motions are 8.75 ± 1.89 s in antagomir group (*n* = 15; red bar) and 1.96 ± 0.47 s in controls (*p* = 0.005, *n* = 15; blue, one-way ANOVA). **(E)** shows whisking durations from the mice in antagomir group (red symbols) and in control (blue). **(F)** shows whisking frequencies in the mice of antagomir group (red symbols) and in control (blues). The frequency and duration of whisker motion are calculated by whisker motion per 0.5 s in all of the time phases. **p < 0.01.

miRNA antagomirs are the chemically modified cholesterol-conjugated single-stranded RNA analogs complementary to specific miRNAs. For instance, miRNA-324-5p antagomir is a RNA analog complementary to miRNA-324-5p. miRNA-133a-3p antagomir is analog to miRNA-133a-3p. Antagomir control is miRNA fragment from *C. elegans*, since it is not functional in mammalians and has been used as the control of miRNA antagomirs (Sun et al., 2013; Wang et al., 2013). Antagomir for miRNA-324-5p (5’Cy3), antagomir for miRNA-133a-3p (5’Cy3) and antagomir control were synthesized by RiboBio Company (Guangzhou, China). The mice were anesthetized by the intraperitoneal injection of urethane (1.5 g/kg) and fixed in a stereotaxic apparatus. The microinjections were fulfilled by imbedding the gage needles (0.3 mm in diameters) on the surface of the barrel cortices and by giving the pressure with the microsyringe (RWD Life Science, Shenzhen, China). One nanomolar antagomirs including anti-miRNA324 and anti-miRNA-133a in a ratio of one to one or antagomir control were dissolved in 2 μl of the ACSF, and were injected into barrel cortices in the mice, in which three time of injections were given to each mouse with 3 days of intervals (i.e., injections on day 1, day 4 and day 7) and each injection was slowly done about 30 min. It is noteworthy that as the retention volume of the injection microneedle is about 1 μl and the delivery velocity of the microinjection is 0.03 μl /min, the local diffusion of the injected solution makes the actual volume into the injection site of the BC being less than 2 μl, such that the side-effect of this microinjection is minimized. The transfected area of the microinjection without injury is showed in Figure 1B. In addition, the transfection rate of miRNA antagomirs and their control in barrel cortices was 50% on average in our study (Supplementary Figure S6), which has been considered in analyses of axon projection and synapse innervation.

### Fluorescence Labeling

The mice were anesthetized by intraperitoneal injections of urethane (1.5 g/kg). In surgical operations, the anesthetic depth was set at the levels where the mice demonstrated lack of reflexes in pinch withdrawal and eyelid blinking. The body temperature was maintained by a heating blanket whose temperature was electronically controlled at 37°C. Ca^2+^-sensitive dye, Oregon Green BAPTA-1-AM (OGB-1, Invitrogen, USA), was used to measure neuronal activities. A craniotomy (2 mm) was made on the skull above barrel cortical areas (1 mm posterior to the Bregma and 3.5 mm lateral to the midline; Paxinos and Watson, 2005). The dura was intact in experiments. The detailed information about surgical operation, dye loading and post-treatment for cell imaging can be referred in our studies (Zhao et al., 2012; Wang et al., 2015).

### Two-Photon Cell Imaging

The anesthetic depth for doing *in vivo* two-photon cell imaging in the mice was set at the moderate reflexes in pinch withdrawal and eyelid blinking, as well as the responses of their whiskers to the test stimuli (Zhao et al., 2012; Wang et al., 2015). Ca^2+^ imaging was done 1 h after dye injections under a two-photon microscope (Olympus FV-1000, Tokyo, Japan), which was equipped by the two-photon laser-beam generator (Mai Tai, Physical Spectrum, USA) and mounted to an upright microscope (Olympus BX61WI) with the water immersion objectives (IR-LUMPLan Fl, 40×, 0.8NA). A wave of two-photon laser beam at 810 nm was given to excite OGB and examine neuronal responses to WS and OS. The emission wavelength was 525 nm for Ca^2+^-binding OGB. Average power delivered to the barrel cortices was less than 75 mW to minimize photo bleach. Whole field images were acquired at 10 Hz of frame rate (256 × 256 pixels). The parameters for photomultiplier tube (PMT) and laser beam were locked in the measurements throughout all experiments to have consistency in data comparison.

Similar to the stimuli in behavioral task, an odor-test pulse toward the noses or mechanical pulses to the whiskers on the contralateral side of two-photon imaged cortices were used to induce cell responses. Stimulus patterns were pair-pulses (OS vs. WS or WS vs. OS) and pulse intervals were 60 s (Wang et al., 2015; Yan et al., 2016).

### Imaging Data Analyses

Cellular Ca^2+^ fluorescence signals in response to stimuli were acquired by Fluoview-10 software (Olympus Inc., Japan) and analyzed in cell bodies by NIH ImageJ and MATLAB (MathWorks). To reduce photon and PMT noise, a median filter (radius, 1 pixel) was used to all images. Ca^2+^ signals were normalized and presented as relative fluorescence changes (ΔF/F). Basal fluorescence (F) was an averaged value before stimuli. Values about ΔF were the differences of Ca^2+^ signal fluorescent strengths between evoked response and basal condition (Zhao et al., 2012). All fluorescence signals were subtracted from the noise signals of unstained blood vessels. Normalized Ca^2+^ signals were smoothed by low-pass Butterworth filter to remove low-level fluctuation and to minimize distortions from Ca^2+^ transient (Yaksi and Friedrich, 2006). The effective signals from each of active cells were judged based on a criterion that their relative fluorescence changes were greater than 2.5 times of standard deviation of baseline values lasting for 500 ms (Wang et al., 2015). The magnitudes of Ca^2+^ transient signals, i.e., activity strength, were measured from the point at 2.5 times of standard deviation of baseline values to their peaks. The durations of Ca^2+^ transient signals were measured as the time from the point at 2.5 times of standard deviation of baseline values in the rising phase to the point at 2.5 times of standard deviation of baseline values in the decay phase. The data were presented as mean ± SEM (Zhang et al., 2012; Zhao et al., 2012; Wang et al., 2015).

With these recorded Ca^2+^ signals, we analyzed their strengths and durations of the barrel cortical neurons in response to WS and OS (Wang et al., 2015). The strength of Ca^2+^ signal was proportional to the spike frequency, and the duration of Ca^2+^ signals was proportional to number of spikes (Petersen et al., 2005; Yaksi and Friedrich, 2006). It is noteworthy that fluorescent signals are measured one time, but not repetitively, in order to prevent their photo bleach and effects on signal strength analyses.

### Neural Tracing and Synapse Formation

The structural connections between cortical regions were traced by injecting pAAV-SynaptoTag-Cherry-GFP (a gift from Dr. Tom Südhof) into the piriform cortex (PC) and by detecting its presence in the BC from C57 Thy1-YFP mice (Zhang et al., 2013) whose glutamatergic neurons were genetically labeled by YFP. The working principle of this AAV was that Synapsin-I promoter initiates the expression of EGFP-synaptobrevin-2 in presynaptic boutons and terminals as well as the expression of mCherry in the neuron (Xu and Südhof, 2013), especially in cell body (Figure 3A). In pAAV injection, the glass pipettes were positioned in the PC (1.0 mm posterior to the Bregma, 3.5 mm lateral to midline and 4.75 mm in depth), based on the map of the mouse brains (Paxinos and Watson, 2005). Two weeks after injecting pAAV into their cortices, the axon projection and synapse formation were analyzed.

**Figure 2:**
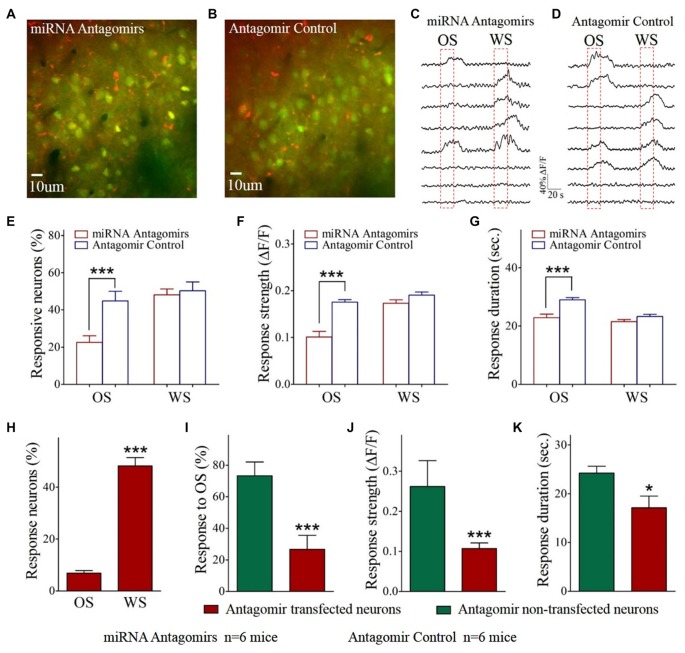
The responses of barrel cortical neurons to OS are downregulated by the antagomirs of miRNA-324-5p/miRNA-133a-3p. **(A)** shows the Ca^2+^ images in the neurons from a mouse in antagomir group. As miRNA antagomir is conjugated by red fluorescent Cy3, yellow cells are thought to be miRNA antagomir transfected neurons. **(B)** shows the Ca^2+^ images in the neurons from a mouse in control. **(C)** illustrates the digitized Ca^2+^ signals from the neurons in response to OS and WS from a mouse of antagomir group. **(D)** shows the digitized Ca^2+^ signals from the neurons in response to OS and WS from a mouse of controls. **(E)** The percentages of the neurons in response to OS are 22.60 ± 3.50% of fluorescence-detected neurons from antagomir group (*n* = 6 mice, red bar) and 44.80 ± 5.20% in control group (*p* = 0.003, *n* = 6 mice; blue). **(F)** To OS-responsive neurons, Ca^2+^ signal strengths are 0.1 ± 0.01 in antagomir group (*n* = 49 cells; red bar) and 0.18 ± 0.005 in controls (***p < 0.001, *n* = 141 cells; blue bar).** (G)** Ca^2+^ signal durations are 22.90 ± 1.20 s in antagomir group (*n* = 49 cells; red bar) and 29.0 ± 0.80 s in controls (***p < 0.001, *n* = 141 cells; blue bar).** (H)** shows the percentages of antagomir-transfected neurons in response to the OS (8 out of 116 cells) and to the WS (56 out of 116) in antagomir group.** (I)** shows the percentages of the neurons in response to the OS that are antagomir-transfected (red-filled bar) and non-antagomir-transfected (green-filled) from antagomir group mice. **(J)** Ca^2+^ signal strengths in response to OS are 0.26 ± 0.06 in non-transfected neurons (*n* = 41 cells, green bar) and 0.11 ± 0.01 in antagomir-transfected neurons (***p < 0.001, *n* = 8 cells; red) from antagomir group mice. **(K)** Ca^2+^ signal durations in response to OS are 24.25 ± 1.39 s in non-transfected neurons (*n* = 41 cells; green bar) and 17.10 ± 2.40 s in antagomir-transfected neurons (*p* = 0.03, *n* = 8 cells; red) from antagomir group mice. *p< 0.05.

**Figure 3:**
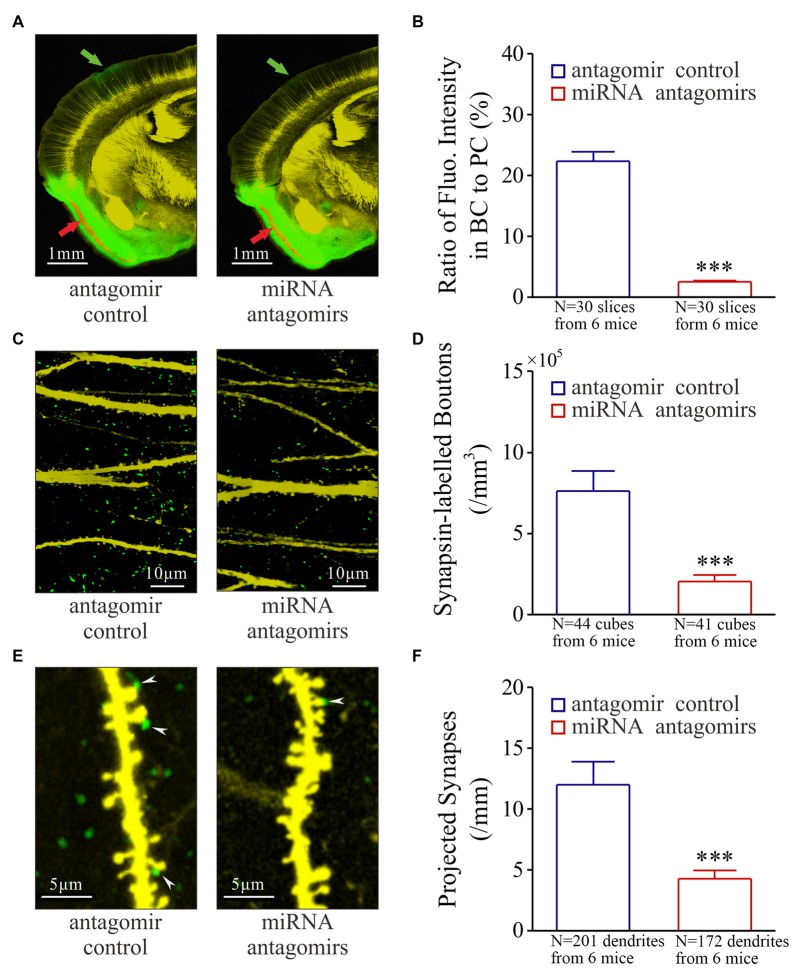
Synaptic innervations from the piriform cortex (PC) to the barrel cortex (BC) after their co-activation are downregulated by the antagomirs of miRNA-324-5p/miRNA-133a-3p. Neural tracing is done by injecting pAAV-SynaptoTag-Cherry-GFP into the PC and by detecting its presence in the BC. **(A)** illustrates neural tracings in the mice from control group (left panel) and antagomir group (right). Red arrow shows injection site and green arrow is the projected site. **(B)** Based on the ratio of green fluorescent intensity in BC to red fluorescent intensity of neuronal somata in PC, the strengths of the projected axons are 2.50 ± 0.20% in antagomir group (red bar, *n* = 30 slices from 6 mice) and 22.40 ± 1.50% in control (blue bar; ***p < 0.001, *n* = 30 slices from 6 mice). **(C)** shows synapsin I-labeled boutons in the barrel cortices from control mice (left panel) and antagomir groups (right panel). **(D)** The densities of synapsin I-labeled boutons are 2.0 ± 0.4 × 10^5^ per mm^3^ in antagomir group (red bar; *n* = 41 cubes) and 7.60 ± 1.27 × 10^5^/mm^3^ in controls (blue; ***p < 0.001, *n* = 44). **(E)** shows synapse contacts (white arrows) between presynaptic boutons (green) and postsyanptic spines (yellow) on dendrites (per mm) in the barrel cortices from mice in control (left panel) and antagomir groups (right). **(F)** The number of new synapses on apical dendrties are 4.70 ± 0.70 per mm in antagomir group (red bar; *n* = 172 dendrites) and 11.90 ± 1.90 per mm in control group (blue; ***p < 0.001, *n* = 201).

The mice were anesthetized by intraperitoneal injections of sodium pentobarbital, and perfused by 4% paraformaldehyde in 0.1 M phosphate buffer solution (PBS) into left ventricle until their bodies were rigid. The brains were quickly isolated and fixed in 4% paraformaldehyde PBS for additional 24 h. The cerebral brains were sliced in a series of coronal sections at 100 μm. These slices were rinsed by PBS for three times, air-dried and cover-slipped. In order to clearly show three-dimension images for new synapses in the BC, we placed the brain slices into a solution (Sca/eA2) for a few hours to make them transparent (Hama et al., 2011).

The images to show synaptic contacts between GFP-labeled axon boutons and YFP-labeled glutamatergic neuron spines in the barrel cortices were used to count the new synapses. The images for YFP-labeled glutamatergic neurons and GFP-labeled axonal boutons in cortical layers II–III were photographed under this confocal microscopy with oil lens (Plan Apo VC 60×, 1.4NA; Nikon A1R plus). The excite wavelength was 488 nm for GFP and YFP. Although the peaks of the GFP and YFP emission wavelengths are closely at 510 nm and 525 nm, respectively, we scanned the images of these neurons by setting up the optical grating in 505–515 nm for GFP and the optical grating in 545–555 nm for YFP to separate their fluorescent images. These images were merged to construct the newly formed synapses. Although we have reported the functional connection from the PC to the BC in CR-formation mice (Wang et al., 2015), it is noteworthy that these synaptic contacts are examined by seeing their functions in spines/boutons and their labeling with synapse-marker proteins. In this regard, we would call these synapse contacts as “presumed new synapses”, though they are stated as “newly formed synapses” in the text of our article. In the confocal imaging, the resolution was 0.05 μm per pixel, the minimal pixels for the measured spines were at least 9–10 in the line. The structure and the density of the newly formed synapses were analyzed in layers II–III of the barrel cortices by applying softwares ImageJ (version. 1.47; The National Institute of Health, MD, USA) and Imaris (version 7.2.3; Bitplane, England).

In the analyses of the dendritic spines, axon terminals and the synapses, we defined the contacts as the synapses based on the separations between presynaptic and postsynaptic identities less than 0.1 μm. We calculated synaptic contacts per millimeter dendrites. Moreover, as the YFP did not label all of the glutamatergic neurons due to the weak Thy1 promoter, synapsin-GFP-labeled presynaptic boutons might innervate the spines on non-YFP glutamatergic neurons, such that the densities of GFP-labeled boutons per mm^3^ were also calculated to assess the number of new synapses.

### Dual Luciferase Reporter Assay

The entire procedure of Dual Luciferase Reporter Assay was given in our previous publication (Ma et al., 2016). The 3′-untranslated region (UTR) sequence of *Ttbk1* was amplified and fused into the *XhoI* and *NotI* sites of the dual luciferase vector psiCHECK2, a generous gift from Dr. Xue (Institute of Biophysics, Chinese Academy of Sciences). The site-mutation of the detected miRNA targeting site of 3′-UTR fragment was constructed based on a guideline of QuikChange Lighting Site-Directed Mutagenesis Kit (Stratagene, La Jolla, CA, USA). miRNA mimics and their negative control (NC) were synthesized by Guangzhou Rui Bo Biological Technology Co. Ltd., China. Primer sequences are listed in Supplemenatry Table S1. For the luciferase reporter detection, HEK293T cells were planted in RPMI media containing 10% fetal bovine serum at 5 × 10^4^ cells per well in 24-well plates. After 24 h, these cells were co-transfected with 20 ng psiCHECK2-3’UTR wild-type or mutant reporter plasmids. In the meantime, these wells of cell culture were added by 50 nM of miRNA mimic or miRNA-NC by using Lipofectamine 2000 transfection reagent (Invitrogen, Carlsbad, CA, USA). The activities of firefly and Renilla luciferases were assessed after 48 h by using Dual-Glo^®^ Luciferase Assay System (Promega, Cat. E2920, USA), based on the manufacturer’s protocols. A ratio of Renilla luciferase activity to firefly luciferase activity was used to analyze the level of mRNA expressions regulated by miRNAs, such as mRNA downregulation or upregulation (Parsons et al., 2000). Each treatment was performed in the triplicates from three independent experiments.

### Western Blot

Barrel cortical tissues from mice whose barrel cortices received the injections of miRNA-324-5p/miRNA-133a-5p antagomirs or antagomir control were gently washed three times in ice-cold PBS and then placed in 1 ml of RIPA Lysate buffer with PMSF (Beyotime Biotechnology, China) for their homogenizations. The homogenized tissues were removed into a new EP tube (1.5 ml), kept at 4°C in a refrigerator for 30 min, and centrifuged at 12,000 *g*/min for 15 min. The concentration of total proteins in supernatant liquid was measured by using the protein assay based on manufacturer instruction. Fifty micrograms of total proteins per sample was then resolved in 10% sodium dodecyl sulfate polyacrylamide gel electrophoresis (SDS-PAGE). After SDS-PAGE, proteins were electrically transferred onto nitrocellulose membrane. The membrane was incubated by blocking solution (1× TBS; 0.1% Tween 20; 5% non-fat milk) at room temperature (25°C) for 2 h, and then incubated overnight by a primary antibody (1:2000 in dilution) of Ttbk1 (ab103944, Abcam) or by primary antibody (1:1000 in dilution) of β-actin (AC004, ABclonal Technology) in 5% milk in TBST. After incubations with their corresponding secondary antibodies conjugated with peroxidase (Beyotime Biotechnology, China), the bands of these proteins were visualized by using the enhanced chemiluminescence ECL Plus immunoblotting detection system (Climx Science Instruments Co. Ltd., China). The protein bands corresponding to the expected size were selected and read by using this computerized scanner. The pixel density in each of these bands was determined by this computer after the background was corrected, which were used to quantify for the relative level of these proteins. The optical densities of each band relative to measured values of β-actin bands were determined using ImageJ software (Zhu et al., 2017).

### Statistical Analyses

The paired *t*-test was used in the comparisons of the experimental data before and after associative learning as well as the neuronal responses to WS and odorant stimulus in each of the mice. One-way ANOVA was used for the statistical comparisons in the changes of neuronal activity and morphology between the miRNA antagomir and antagomir control groups. It is noteworthy that the behavioral studies and the data analyses are conducted by different individuals.

## Results

In the present study, we aim to study the causal relationship from new synapse innervations and associative memory cell recruitment to associative memory as well as to examine their molecular mechanisms by manipulating epigenetic process. To investigate the roles of epigenetics-mediated events, such as miRNAs, in associative memory cell recruitment, synapse innervation and associative memory, we have injected the antagomirs of miRNA-324-5p and miRNA-133a-3p or antagomir NC into mouse barrel cortices (Figures 1A,B). The selection of these two anti-miRNAs was based on our observation that miRNA-324 and miRNA-133a, which presumably regulate axon growth and synapse formation, were upregulated in barrel cortices from mice that expressed odorant-induced whisker motion (Yan et al., 2016). In some mice from these two groups, the injection of pAAV-SynaptoTag-mCherry-GFPs (Xu and Südhof, 2013) into the piriform cortices and the detection of its presence in the barrel cortices were conducted to examine the effect of anti-miRNA on synapse formation. These mice were subsequently trained by pairing the OS and the WS for 10 days (Wang et al., 2015; Gao et al., 2016).

After the training in the mice, the OS-test was given to their noses. The mice that have received antagomir control express whisker motion in response to the OS (Figure 1C). This odorant-induced whisker motion appears to be impaired in the mice that have received the antagomirs of miRNA-324 and miRNA-133a (Figure 1C). Latencies from the OS onset to whisker motion is prolonged in antagomir group, compared with antagmir control (Figure 1D). Whisking durations (Figure 1E) and frequencies (Figure 1F) in response to the OS are lowered in antagomir group than antagomir control. In addition, the changes in whisking amplitude and bouts duration are given in Supplementary Figure S1. Thus, anti-miRNA-324 and anti-miRNA-133a downregulate odorant-induced whisker motion, i.e., miRNA-324 and miRNA-133a are required for associative memory.

The influence of anti-miRNAs on the recruitment and activities of associative memory cells was examined in the barrel cortices of WS/OS-trained mice by using two-photon cell Ca^2+^ imaging, which had been injected with the antagomirs of miRNA-324 and miRNA 133a (Figure 2A) or antagomir control (Figure 2B). Figures 2C,D show the digitized traces of cellular Ca^2+^ signals in response to the WS vs. the OS from a mouse in antagomir group and from a mouse in antagomir control group. The proportions of the neurons in response to the OS are significantly lower in antagomir group than antagomir control (Figure 2E), implying that anti-miRNA-324/-133a attenuate associative memory cell recruitment. To OS-responsive cells, Ca^2+^ signal strengths (Figure 2F) and durations (Figure 2G) are significantly lower in antagomir group than antagomir control. However, Ca^2+^ signals in response to the WS are not statistically different between antagomir group and antagomir control group (Figures 2E–G). Our results indicate that anti-miRNA-324 and anti-miRNA-133a downregulate the conversion of barrel cortical neurons into associative memory cells, as well as their activity strength and duration.

This indication should be further tested in barrel cortical neurons that are transfected vs. non-transfected by miRNA antagomirs in mice whose barrel cortices receive the injection of miRNA antagomirs. In barrel cortices, red fluorescent-labeled neurons were thought to be antagomir-transfected neurons, or vice versa, since miRNA antagomirs had been conjugated with Cy3. The proportions of miRNA antagomir-transfected neurons are 48.2 ± 5.2% in response to the WS and 6.9 ± 1.0% in response to the OS (Figure 2H), indicating the less effect of anti-miRNA on WS-responses. To OS-responsive cells, 73.3 ± 8.8% of them appear to have no antagomir-transfection and 26.7 ± 8.8% of them antagomir-transfection (Figure 2I). Ca^2+^ signal strength and duration in response to the OS are significantly lower in antagomir-transfected cells than non-transfected cells (Figure 2J,K). These parameters are not changed in the cells transfected by antagomir control (Supplementary Figure S2). This result supports the indication that anti-miRNA-324 and anti-miRNA-133a downregulate the recruitment of associative memory cells and their activities by reducing their responses to the OS but not the WS. In other words, miRNA-324 and miRNA-133a are required for recruiting associative memory cells.

The recruitment of associative memory cells in the BC may be driven by the synapse innervations from the PC (Wang et al., 2015; Gao et al., 2016), which we have tested by the injection of pAAV-SynaptoTag-Cherry-GFP into the PC (red arrow in Figure 3A) and the detection of its presence in the BC (green, Supplementary Figure S3). After the WS/OS-paired training in these mice, axon projections from the PC to the BC are detected in antagomir control, but less in antagomir group (Figure 3A). The strength of the projected axons is statistically lower in antagomir group than antagomir control group (Figure 3B). Synapsin I-labeled boutons in the BC are statiscially lower in antagomir group than antagomir control group (Figures 3C,D). The synapses that are the contacts between GFP-labeled presynaptic boutons and YFP-labeled postsynaptic spines on glutamatergic neurons in the BC appear low in antagomir group, compared to those in antagomir control group (Figure 3E), which is statistically different (Figure 3F). In addition, the changes in spine density and volume are showed in Supplementary Figure S4. Thus, anti-miRNA-324 and anti-miRNA-133a downregulate synapse innervations from the PC to the BC during associative memory. That is, miRNA-324 and miRNA-133a are required for new axon projection and synapse formation.

Taken these results together, we suggest that there are the causal relationships among synapse formation, associative memory cell recruitment and associative memory, in which the epigenetic molecular events triggered by miRNA-324 and miRNA-133a play critical roles in these processes.

**Figure 4:**
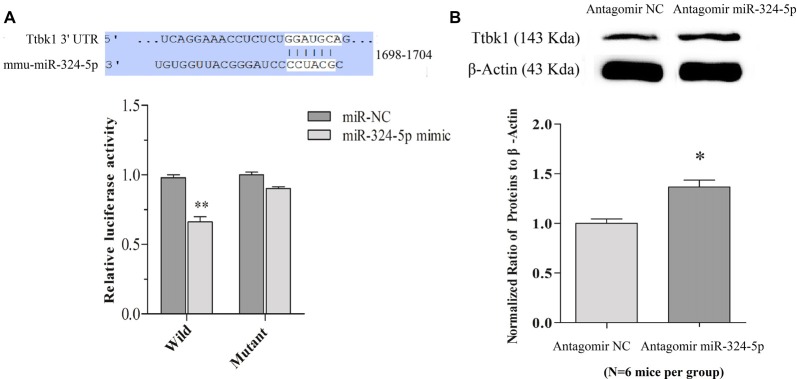
miRNA-324-5p acts onto *Ttbk1* and downregulates Tau-tubulin kinase-1. **(A)** Luciferase reporter assay is done by the co-transfection of luciferase reporter containing wild or mutant 3′-untranslated region (UTR) of *Ttbk1* with miRNA-324-5p mimic or its negative control (NC) into HEK293T cells. Luciferase activity was read 48 h after co-transfection. Top panel shows the predicted interaction between wild-type 3′-UTR of *Ttbk1* and miRNA-324-5p. The relative activities of luciferase reporter for *Ttbk1* 3′-UTR are 1.0 ± 0.05 in the group of adding NC (dark-gray bar) and 0.66 ± 0.07 in the group of adding miRNA-324-5p mimic (*p* = 0.006, *n* = 24; light-gray bar). The relative activities of luciferase reporter for 3′-UTR of *Ttbk1* with mutant miRNA-324-5p binding site are 1.0 ± 0.06 in the group of NC (dark-gray bar) and 0.92 ± 0.05 in the group of miRNA-324-5p (light-gray). The ratio for NC is normalized as 1.** (B)** The expression of Tau-tubulin kinase-1 was studied by western-blot. The ratios of Ttbk1 to β-actin are 1.0 ± 0.1 in antagomir control mice (light-gray bar, *n* = 6) and 1.37 ± 0.07 in miRNA-324-5p antagomir mice (dark-gray, *p* = 0.02, *n* = 6). The ratios for control mice are normalized as 1. *p < 0.05; **p < 0.01.

mRNA *Ttbk1* that translates Tau-tubulin kinase-1 is a predicted target of these miRNAs. This brain-specific kinase phosphorylates microtubule-associated protein Tau, leading to its aggregation (Sato et al., 2006; Xu et al., 2010). Taken this with our studies that miRNA-324 is upregulated (Yan et al., 2016) and Ttbk1 is downregulated (Supplementary Figure S5) in barrel cortices of CR-formation mice, we have examined whether miRNA-324-5p directly acts on *Ttbk1* by dual luciferase report assay. Luciferase reporter plasmids including the wild-type or mutant 3′-UTRs of predicted miRNA-324-5p binding site in *Ttbk1* (top panel in Figure 4A) were transfected into HEK293T cells. After miRNA-324-5p or its NC is added into cell cultural dishes for 24 h, the relative activity of luciferase reporter for *Ttbk1* 3′-UTR is significantly inhibited by miRNA-324 and this inhibition is reversed by mutating miRNA-324-5p binding site (Figure 4A). That is, *Ttbk1* is a target of miRNA-324-5p.

The influence of anti-miRNAs on the expression of Ttbk1 protein was examined by western-blot. The proteins were harvested from the barrel cortices that had received the injection of miRNA antagomirs or their controls. The levels of Ttbk1 appear higher in antagomir group than in antagomir control, which is statistically different (Figure 4B). Memory cell recruitment and synapse formation in associative memory are influenced by downregulating Tau-tubulin kinase-1.

## Discussion

Pairing whisker and odor signals in the mice leads to odorant-induced whisker motion alongside natural whisker-induced whisker motion, i.e., cross-modal associative memory. After receiving new synapse innervations from the axons of co-activated piriform cortical neurons, the barrel cortical neurons are recruited to be associative memory cells that encode the newly learnt odor signal alongside the innate whisker signal. These processes are attenuated by anti-miRNA-324 and anti-miRNA-133a (Figures 1–3). By studying a causal relationship between anti-miRNA and attenuated cellular events, our study indicates that epigenetic-regulated processes induce new synapse innervation, associative memory cell recruitment and associative memory. The associated activations of the barrel and piriform cortices by pairing whisker and odor signals produce their mutual synapse innervations and associative memory cells for the storage of associated signals. Our data reveals a novel mechanism, i.e., new synapse innervation and associative memory cell recruitment primarily in the sensory cortices for associative memory, beyond previous focuses in synaptic plasticity.

**Figure 5:**
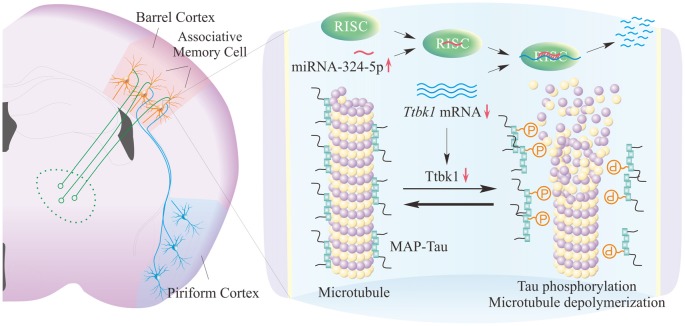
miRNA-324-reguated Ttbk1 presumably plays a crucial role in the recruitment of synapse innervation and associative memory cells in barrel cortices. Left panel shows the synapse innervation on barrel cortical neurons from piriform cortical neurons and the recruitment of associative memory cells in the BC. Right panel shows the potential molecular mechanisms underlying the recruitments of synapse innervation and associative memory cells. The upregulated miRNA-324 downregulates mRNA *Ttbk1* and protein Ttbk1, which reduces the phosphorylation of microtubule-associated protein Tau (MAP-Tau) by shifting the balance between MAP-Tau phosphorylation and dephosphorylation toward dephosphorylated MAP-Tau. These dephosphorylated Tau can stabilize microtubules and facilitate axon growth from the PC as well as synapse and associative memory cell recruitments in the BC.

In our study, miRNA-324 acts and downregulates mRNA *Ttbk1* that guides the expression of Tau-tubulin kinase-1, which is supported by the data of dual luciferase report assay and western-blot in Figure 4. This kinase is able to phosphorylate microtubule-associated protein Tau (Sato et al., 2006; Xu et al., 2010). Phosphorylated Tau proteins induce their aggregations and destabilize microtubules, which lead to microtubule depolymerization, axon degeneration and spine deformation (Sánchez et al., 2000). The downregulation of *Ttbk1* by the upregulated miRNA-324 may shift the balance between axon prolongation and retardation toward axon growth from the PC to the BC, and trigger axon terminals to make new synapses onto barrel cortical neurons. These new synapses may drive these barrel cortical neurons to be recruited as associative memory cells (Figure 5).

The cellular targets of miRNA-mediated epigenetic process for associative memory include the following. Both presynaptic axon boutons (Figure 3) and postsynaptic spines (Supplementary Figure S4) are upregulated in their densities and size, which influence the chance to form new synapse innervations. The recruitment of associative memory cells is upregulated, since anti-miRNA-324/133a reduces the number of associative memory cells as well as their response strength and duration to the new odor signal (Figures 2E–K). As miRNA-324/133a are predicted to regulate the axon prolongation and synapses based on bioinformatics (Yan et al., 2016), our results *in vivo* about axon prolongation and synapse formation are consistent with molecular profiles.

There are two forms of neural plasticity that are associated with memory formation and cognition, i.e., the recruitments of new synapse innervations and associative memory cells as well as the functional change of synapses and neurons. Plasticity in our study falls into new synapse formation and associative memory cell recruitment that are driven by the association of new and innate signals via miRNA-mediated processes. These associative memory cells are specific for the integration and storage of the newly acquired signal and innate signal. On the other hand, plasticity at those existing synapses in a specific pathway, such as LTP (Bliss and Lomo, 1973) and LTD (Stanton and Sejnowski, 1989), may not be related to the integration and storage of associated signals. These activity-dependent potentiation and depression at the synapses after their formation may work for the process whether their innervated neurons are able to be activated for signal retrieval and memory presentation. In this regard, our study about associative memory cells bring insight into cellular mechanisms for memory formation.

Based on our studies, the characteristics of associative memory cell are proposed below. They are recruited to encode multiple associated signals. They receive multiple synapse innervations from the sensory cortices that are co-activated and encode different signals. Their axons project toward the brain areas related to cognitions, emotions and behaviors for memory retrieval. The recruitment of associative memory cells is initiated by epigenetic-mediated process. The number of recruited associative memory cells is proportional to memory strength and maintenance. The downregulations of the associative memory cells and their new synapse innervations reduce memory capacity (Wang et al., 2015; Gao et al., 2016; Wang and Cui, 2017). The working principle of these associative memory cells in cross-modal associative memory may be based on the facilitation of their activity strengths by newly innervated synapses from the co-activated sensory cortices. For instance, barrel cortical neurons receive synapse innervations from the PC. These synapse activities induced by OS can drive these barrel cortical neurons toward a threshold of firing spikes, and their spikes further activate downstream cortical neurons for whisker motion and other relevant behaviors.

## Author Contributions

ZL, DW, NC, KM, WL and ZS contributed to experiments and data analyses. SC contributed to draw diagram. J-HW worked for the concept, project design and article writing.

## Conflict of Interest Statement

The authors declare that the research was conducted in the absence of any commercial or financial relationships that could be construed as a potential conflict of interest.
