# Supplementary material for: Synapse Innervation and Associative Memory Cell Are Recruited for Integrative Storage of Whisker and Odor Signals in the Barrel Cortex through miRNA-Mediated Processes
Source: Front Cell Neurosci. 2017 Oct 25;11:316. doi: 10.3389/fncel.2017.00316 (PMC5661269; doi:10.3389/fncel.2017.00316)
Supplement: Supplementary file 1 [file Table_1.docx]

| **Name of miRNA** | **The fold changes of miRNA level (log2 CR-formation/ control) in barrel cortex** | **Predicted target genes** | **Functions of target genes**  **(+), upregulation**  **(-), downregulation** | **Actions of miRNA upregulation** |
| --- | --- | --- | --- | --- |
| mmu-miR-150-5p | 1.5357249 | Csnk1d | Excitatory synapses (-) | Upregulate excitatory synapses |
|  |  | Nlgn2 |  |  |
| mmu-miR-324-5p | 2.02819739 | Tet3 |  |  |
| mmu-miR-133a-3p | 2.23751336 |  |  |  |
| mmu-miR-3072-3p | 1.30138072 | Slc32a1 | Inhibitory synapses (+) | Downregulate inhibitory synapses |
| mmu-miR-150-5p | 1.5357249 | Nlgn2 |  |  |
| mmu-miR-324-5p | 2.02819739 | Slc32a1 |  |  |
| mmu-miR-23b-3p | 2.01432593 | Nrxn1 |  |  |
| mmu-miR-133a-3p | 2.23751336 | Iqsec3 |  |  |
| mmu-miR-345-5p | 1.10455068 | Gabra4 |  |  |
|  |  | Rgma | Synapse formation or neuron branching (-) | Upregulate synapse formation or neuron branching |
| mmu-miR-324-5p | 2.02819739 | Ttbk1 | Axon guidance (-) | Upregulate axon and dendrite growth and branching |
| mmu-miR-133a-3p | 2.23751336 | Dyrk2 |  |  |
| mmu-miR-3072-3p | 1.30138072 | Mark2 |  |  |
| mmu-miR-133a-3p | 2.23751336 | Dyrk2 | Cytoskeleton (-) | Stabilize neuron morphology |
| mmu-miR-345-5p | 1.10455068 | Rgma | Spines and dendrites (-) | Stabilizes spines and dendrites |
| mmu-miR-324-5p | 2.02819739 | Ttbk1 |  |  |
| mmu-miR-3072-3p | 1.30138072 | Wnk2 | Channels (+/-) | Upregulate excitatory synapses |
| mmu-miR-150-5p | 1.5357249 | Csnk1d | Neurite outgrowth (+) | Downregulate neuron outgrowth |
| mmu-miR-133a-3p | 2.23751336 | Dyrk2 | Neurite outgrowth (-) | Upregulate neurite outgrowth |
| mmu-miR-133a-3p | 2.23751336 | Dyrk2 | Neuron proliferation (-) | Upregulate neuron proliferation |

**Table S1 MicroRNA’s expression and their roles after associative memory forms**
