# Supplementary material for: Synapse Innervation and Associative Memory Cell Are Recruited for Integrative Storage of Whisker and Odor Signals in the Barrel Cortex through miRNA-Mediated Processes
Source: Front Cell Neurosci. 2017 Oct 25;11:316. doi: 10.3389/fncel.2017.00316 (PMC5661269; doi:10.3389/fncel.2017.00316)

**Supporting Figures to: Frontiers in Cellular Neuroscience**

**Synapse innervation and associative memory cell are recruited for integrative storage of whisker and odor signals in the barrel cortex through miRNA-mediated processes**

**Zhuofan Lei^1,2,3#^, Dangui Wang^2#^, Na Chen^2#^, Ke Ma^1#^, Wei Lu^1,2#^, Zhenhua Song^1^, Shan Cui^2^ and Jin-Hui Wang^1,2,3^***

***1) Qingdao University, School of Pharmacy, 38 Dengzhou, Shandong China 266021; 2) Institute of Biophysics, Chinese Academy of Sciences, Beijing China 100101; 3) University of Chinese Academy of Sciences, Beijing China 100049***

Key words: learning, memory, synapse, memory cell, cortex, microRNA and Ttbk1

Word counts: abstract, 100; text, 4955

# Z Lei, D Wang, N Chen, K Ma and W Lu contribute to this work equally

***Corresponding author:**

Jin-Hui Wang, Ph.D. & MD

Brain and Cognitive Sciences

The Institute of Biophysics, Chinese Academy of Sciences

15 Datun Road, Beijing China 100101

[jhw@sun5.ibp.ac.cn](mailto:jhw@sun5.ibp.ac.cn); 86-10-64888472

**Figures and Figure Legends**

**Figure S1** The effect of miRNA-324/miRNA-133a on odorant-induced whisker motion **A)** shows whisking amplitudes in the mice of antagomir group (red symbols) and in control (blues). **B)** shows bouts durations from the mice in antagomir group (red symbols) and in control (blue). **C)** shows bouts intervals from the mice in antagomir group (red symbols) and in control (blue).


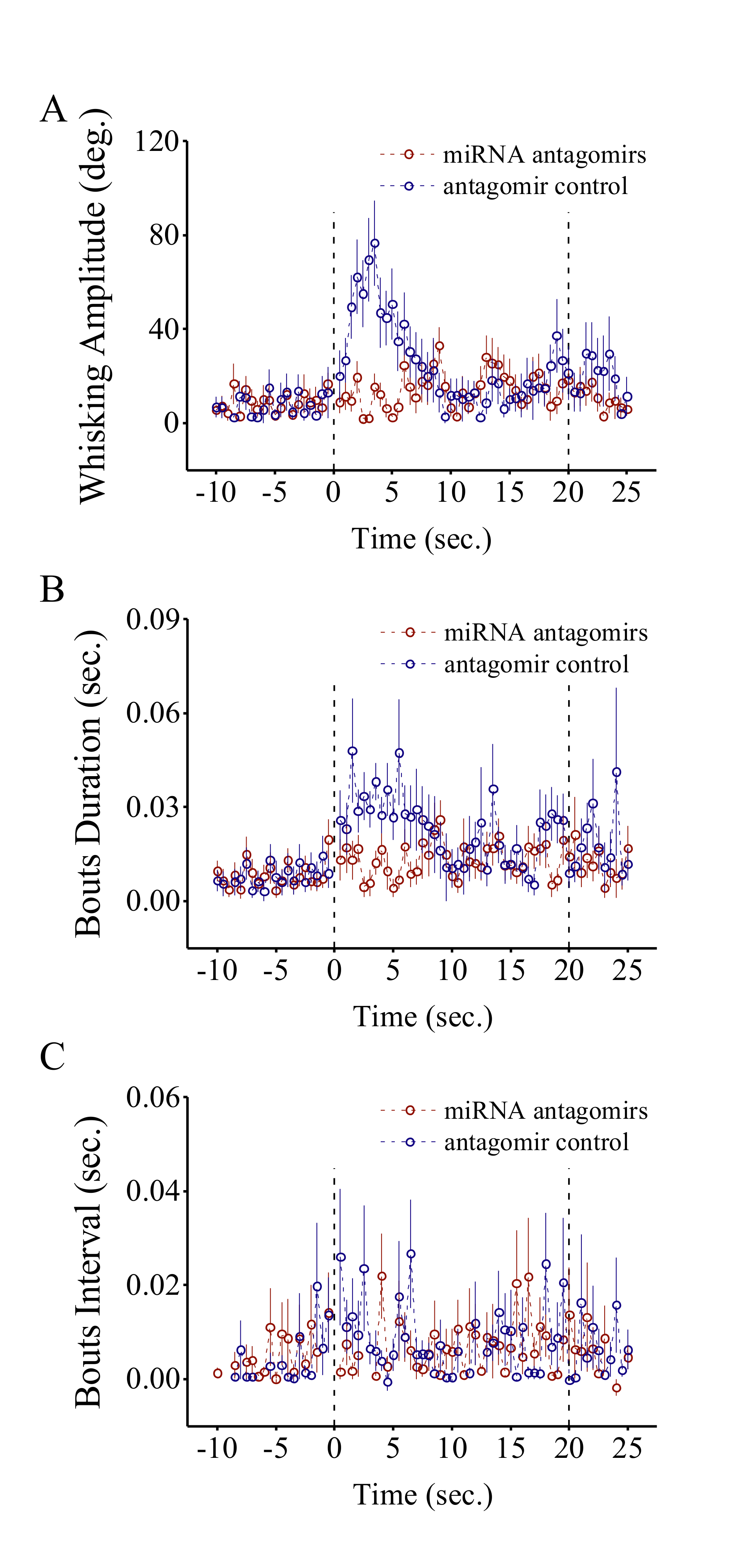


**Figure S2** The responses of the barrel cortical neurons to OS are not affected by the antagomir controls of miRNA-324-5p and miRNA-133a-3p. **A)** shows the percentages of the neurons transfected by antagomir control in response to OS and WS from the mice in the control group. **B)** illustrates the percentages of the neurons in response to OS that are transfected by antagomir control (red bar) and that are not transfected by antagomir control (green bar) from the mice in control group. **C)** shows the OS-response strengths of the neurons transfected by antagomir control (red bar) and non-transfected by antagomir control (green bar) from the mice in control group. **D)** illustrates the OS-response durations of the neurons transfected by antagomir control (red bar) and non-transfected by antagomir control (green) from the mice in control group.

##
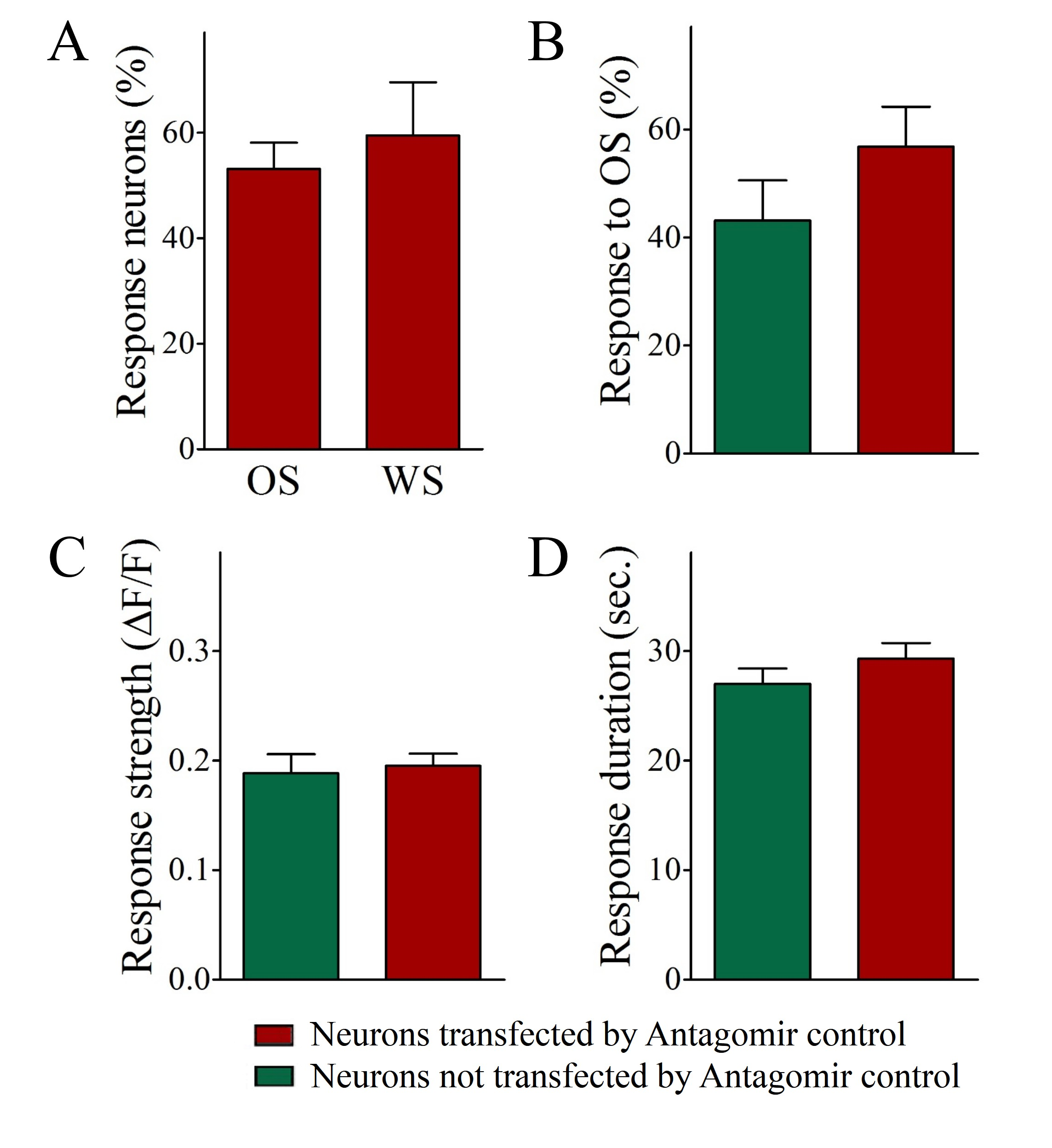


**Figure S3** The injection of pAAV-SynaptoTag-Cherry-GFP into the piriform cortices and the detection of its presence in the barrel cortices were used to examine the effect of anti-miRNA on synapse innervations. **A)** shows a diagram of the injection site (the piriform cortex) of synapsin I-AAV. **B)** The neurons in the piriform cortex are transfected by synapsin I-AAV and the barrel cortex receives the projected axons from the piriform cortex.


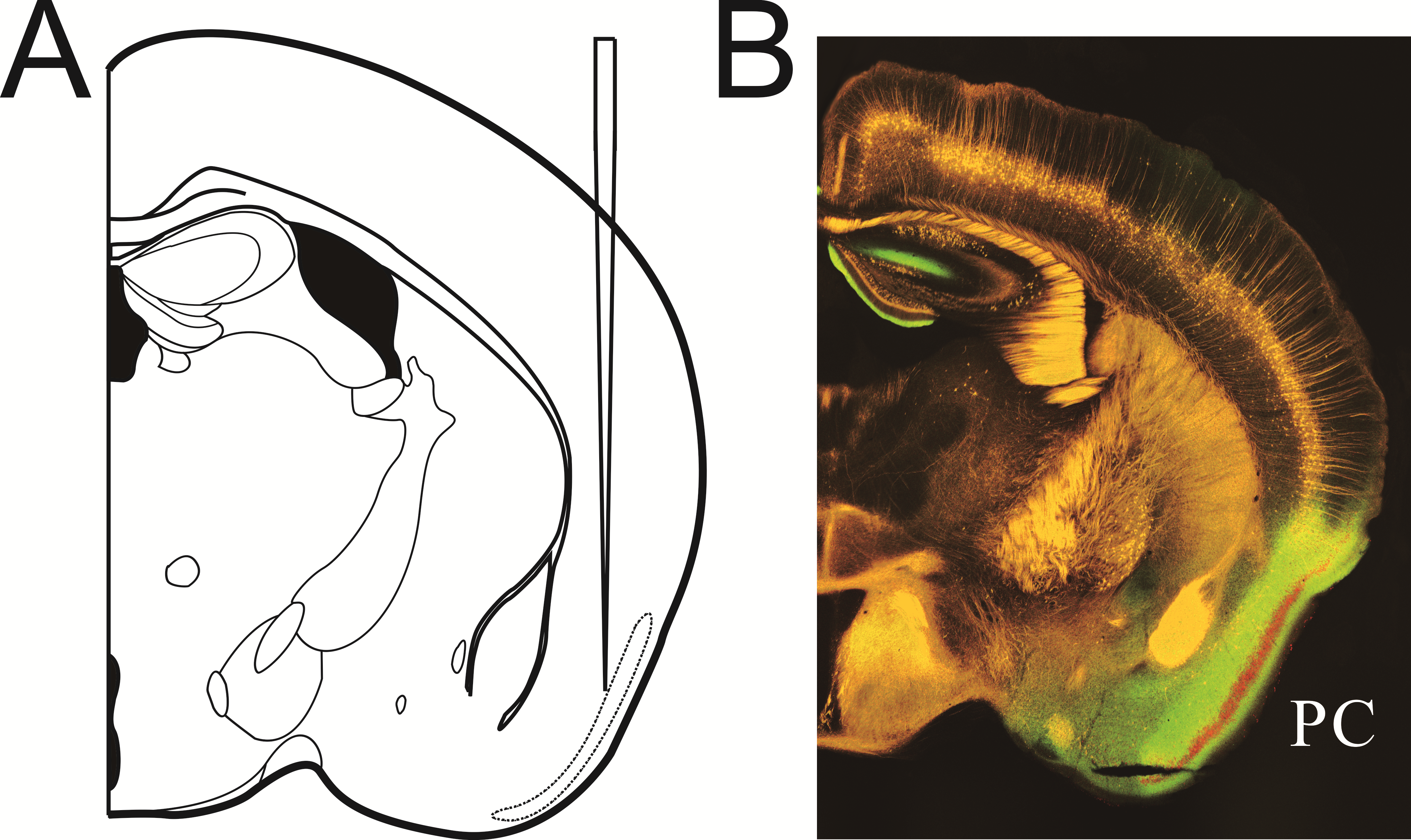


**Figure S4** The effect of miRNA-324/miRNA-133a on spine density and volume **A~B)** illustrates that spine densities are lower in antagomir group (red bar, n=30 slices from 6 mice) than in control (blue bar; p<0.01, n=30 slices from 6 mice). **C~D)** illustrates that spine widths are lower in antagomir group (red bar, n=30 slices from 6 mice) than in control (blue bar; p<0.001, n=30 slices from 6 mice). **E~F)** illustrates there are no changes in spine length neck width in antagomir group (red bar, n=30 slices from 6 mice) versus in control (blue bar; p<0.001, n=30 slices from 6 mice). Two asterisks denote p < 0.01 and three asterisks denote p < 0.001.

##
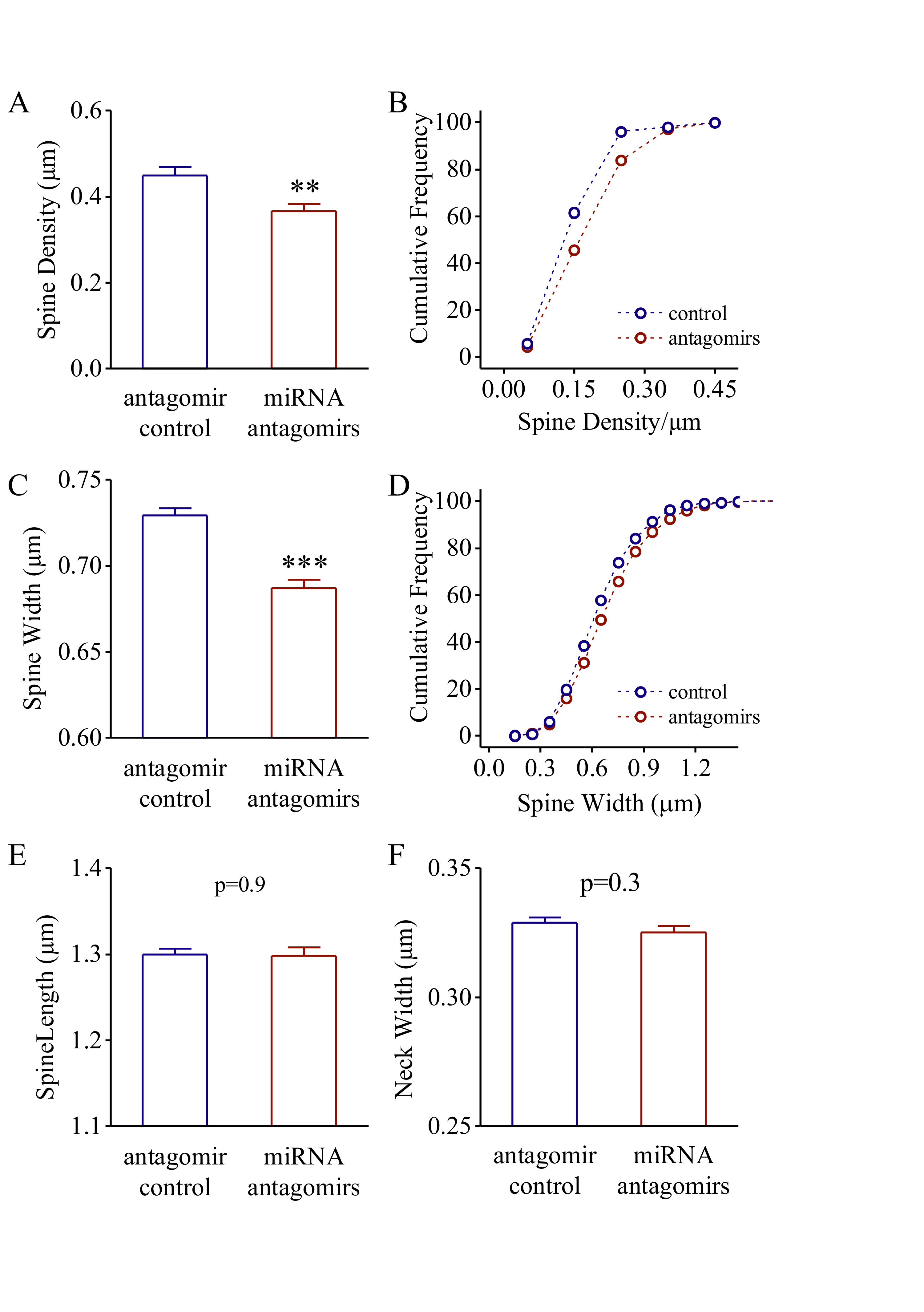


**Figure S5** Ttbk1 and Tet3 proteins in the barrel cortex are downregulated in CR-formation mice trained by pairing WS, OS and TS. **A)** shows the images about Tet3 (top panels), Ttbk1 (middle panels) and β-actin (bottom panels) from western-blot experiments, in which the tissues were from barrel cortices of CR-formation mice (right panels) versus unpaired control mice (left panel). **B)** shows the statistical analysis of Tet3 and Ttbk1 expressions from barrel cortical tissues from CR-formation mice (white bars) and unpaired mice (gray bars). Two asterisks denote p<0.01 and three asterisks denote p<0.001.


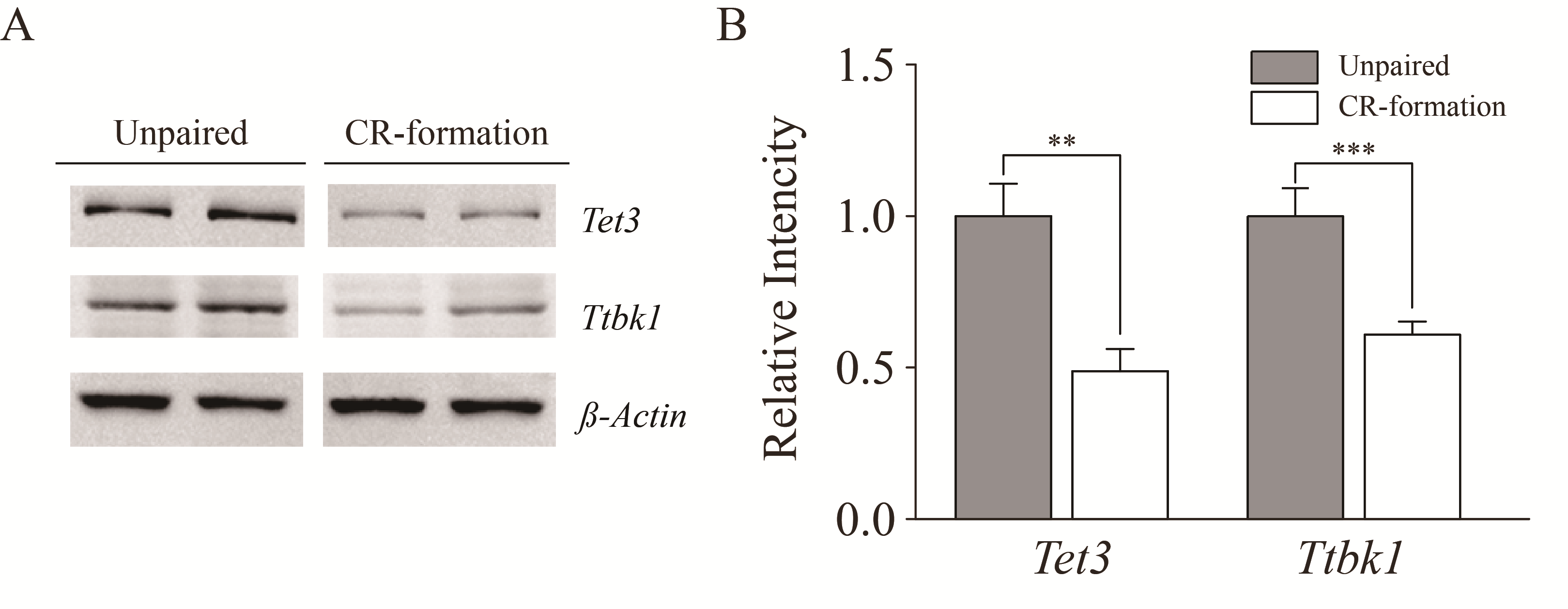


**Figure S6** The transfection rate of miRNA antagomirs and their controls after they are injected into barrel cortices for two weeks, which are accounted based on the ratios of transfected cells by Cy3-conjugated miRNA antagomirs or antagomir controls to Dapi-labeled cells.


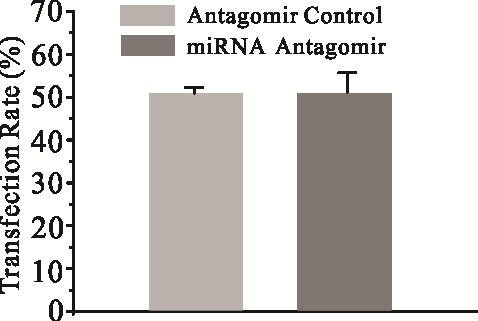


**Figure S7** The influences of miRNA antagomirs on Ttbk1 expression in original images.


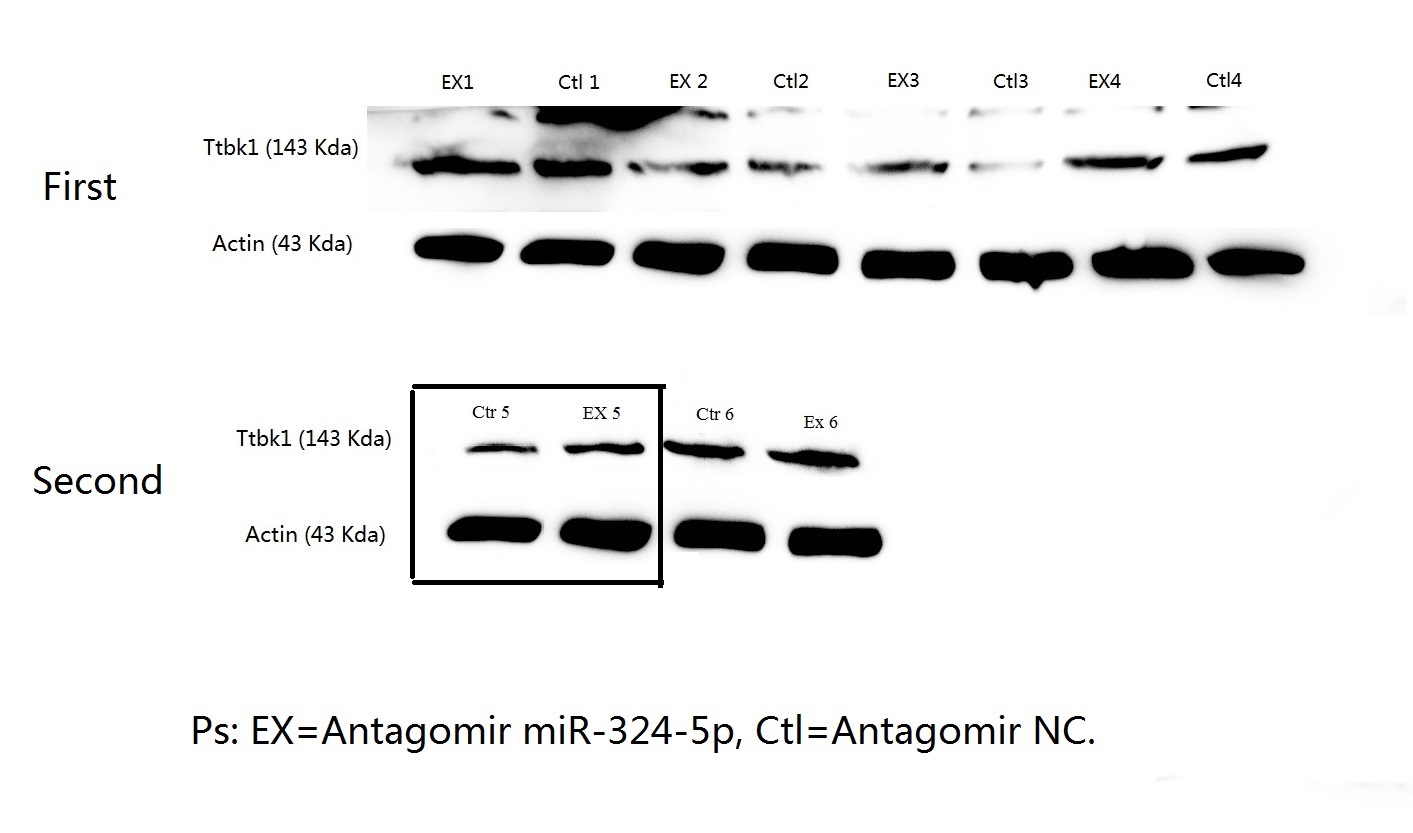

Supplement: Supplementary file 2 [file Data_Sheet_1.doc]
